# Effect of Core Stabilizing Training on Young Individuals Presenting Different Stages of Degenerative Disc Disease—Preliminary Report

**DOI:** 10.3390/ijerph18073499

**Published:** 2021-03-28

**Authors:** Tomasz Kuligowski, Błażej Cieślik, Natalia Kuciel, Agnieszka Dębiec-Bąk, Anna Skrzek

**Affiliations:** 1 Faculty of Physiotherapy, University School of Physical Education in Wroclaw, 51-612 Wroclaw, Poland; tomasz.kuligowski@awf.wroc.pl (T.K.); agnieszka.debiec-bak@awf.wroc.pl (A.D.-B.); anna.skrzek@awf.wroc.pl (A.S.); 2 Faculty of Health Sciences, Jan Dlugosz University in Czestochowa, 42-200 Czestochowa, Poland; 3 Department and Division of Medical Rehabilitation, Wroclaw Medical University, 50-367 Wroclaw, Poland; natalia.kuciel@umed.wroc.pl

**Keywords:** hernia, manual therapy, traction, disc disease, core stability

## Abstract

The aim of this study was to assess the efficacy of stabilizing training for the deep core muscles of the lumbar spine in subjects with degenerative disc disease. This study was conducted on 38 participants. The participants were divided into two groups: the extrusion group (EXT, *n* = 17) and the protrusion group (PRO, *n* = 21). All the subjects underwent a four-week-long core stability exercise-based treatment (five sessions/week). Clinical outcome measures were assessed pre-intervention (pre), post-intervention (post) and four weeks after the intervention (follow-up). The primary outcome measures were the spinal range of motion (ROM; Spinal Mouse^®^ device) and the Oswestry Disability Index (ODI). In the PRO group, the ROM decreased from 88.52° pre-intervention to 83.33° post-intervention and to 82.82° at follow-up (*p* = 0.01), while the ODI decreased from 16.14 points pre-intervention to 6.57 points post-intervention, with 9.42 points at follow-up (*p* < 0.01). In the EXT group, the ROM decreased from 81.00° pre-intervention to 77.05° post-intervention, then increased to 77.94° at follow-up (*p* = 0.03), while the ODI decreased from 22.58 points pre-intervention to 15.41 points post-intervention and to 14.70 points at follow-up (*p* < 0.001). Although the stabilizing exercise sessions improved the clinical outcomes in each group, we cannot make conclusions as to whether the type of intervertebral disc damage significantly affects the results of stabilizing exercise-based treatment.

## 1. Introduction

Degenerative disc disease (DDD) is one of the main causes of low back pain and contributes to the socioeconomic problems [1,2]. It is caused by damage to the tissue forming the intervertebral disc. Despite little to no innervation of the intervertebral disc, its pathology negatively affects many other structures in its proximity, such as the highly innervated posterior longitudinal ligament, spinal dura mater and nerve roots, which can cause a number of pathological conditions leading to pain [3].

Degenerative disc disease is a progressive condition. Depending on the reason for dysfunction, various classifications are used to describe the level of advancement [4]. The scientific literature on DDD commonly distinguishes between two types of the disease, protrusion and extrusion of the intervertebral disc, as approved by the American Society of Neuroradiology (ASNR). The clinical parameters of individuals with symptoms of a protruded or extruded lumbar disc are known to vary depending on the progression level of the disease and are usually recognized as acute, subacute and chronic phases depending on the symptoms, such as pain, functional outcome and ability to work [5].

Contemporary methods for imaging and assessment of the activation of desired muscle groups, such as ultrasonography or electromyography (surface EMG, SEMG; intramuscular EMG, IEMG), make it possible to determine the involvement of certain muscles in the given movement sections or their exclusion from certain activities [6]. Thus, it is possible to determine the exact function of a particular muscle group and administer targeted therapy, which contributes to achieving maximum recovery of the patient. Spinal stability is achieved through cooperation of the neural, muscular and skeletal systems. The main muscle structures responsible for stabilization of the lower back are the lumbar multifidus muscle and the deep abdominal muscles, particularly the transverse abdominal muscle; thus, targeted training of these groups is implemented in case of their dysfunction [7].

Stabilizing training is a form of conservative treatment for lumbar pain alongside manual treatment or techniques in the fields of chiropractic and physiotherapy [8,9]. Conservative treatment methods usually constitute the first-line therapy for low back pain. In case of no improvement, those might also be a decisive tool within the need and timing of the surgery in the light of poor correlation between the radiological and clinical outcomes [10,11]. It is thought that proper stabilization of this region of the body is crucial for coping with pain [12,13,14]. Although studies continue to examine the effectiveness of stabilization training, they primarily relate to chronic nonspecific low back pain [14,15]. Moreover, there is a lack of studies in the literature that attempted assessing the efficiency of physiotherapeutic treatment with regard to stabilization of the deep core muscles in young people with a protruded or extruded disc. We hypothesized that the core stabilizing training would have an effect on both types of the disease within the functional outcome but the time of the patient reaction can be different. Therefore, the aim of the study was to assess the effectiveness of core stability exercise in young patients with lumbar disc protrusion or extrusion.

## 2. Materials and Methods

### 2.1. Participants

The study included 42 experimental subjects (21 men and 21 women) with DDD and was designed as a non-randomized before-and-after study without a control group. The participants were recruited from Wroclaw Medical University and a private physiotherapy clinic. The recruitment was took place from January to December 2016. All the subjects underwent an imaging examination in the form of MRI, the results of which were interpreted by a radiologist. All the procedures followed were in accordance with the ethical standards of the responsible committee on human experimentation (institutional and national) and with the Declaration of Helsinki 1975 as revised in 2008. The research study protocol was approved by the IRB of the authors’ affiliated institutions and was registered at ClinicalTrials.gov (accessed on 25 February 2021) (NCT04119466) in June 2019. All the subjects received detailed information about the research project and gave written consent to participate in the study. They were also informed about the possibility of withdrawal from the study at any time without any consequences.

The inclusion criteria included age between 20 and 35 years, DDD in the lumbar region of the spine confirmed by MRI and subacute stage of the disease. The exclusion criteria included advanced deformation changes of the spine, previous fracture of the spine, neurological deficits in the lower limbs or pelvis, tumors, spondylolisthesis, transitional vertebra, rheumatic disease or mental health disorders.

After enrollment, the damage to the intervertebral disc was classified as protrusion or extrusion based on the recommendations of the ASNR, with 21 patients included in the group with intervertebral disc protrusion (PRO) and 21—in the group with intervertebral disc extrusion (EXT).

### 2.2. Intervention

All the subjects underwent core stability exercise sessions over a period of four weeks (20 sessions in total) based on the protocol proposed by Richardson [16]. The exercise was conducted from Monday to Friday and included activation of the lumbar multifidus muscle (m. multifidus) and the transverse abdominal muscle (m. transversus abdominis). One session comprised four exercises in which the patient was instructed to perform pelvic tilts (draw-in) with simultaneous full exhalation, thus activating the aforementioned muscle groups in different positions: (a) prone, (b) supine with lower extremities flexed, (c) quadruped and (d) standing with the back against the wall. The subjects performed three sets consisting of 10 repetitions, each of which lasted approximately 10 s. After this treatment period, the subjects were asked to refrain from any other form of exercise until the third examination (follow-up).

### 2.3. Outcome Measures

Clinical outcome assessment was performed at three timepoints during the study: prior to initiation of the exercise (pre-intervention, week 0), following the conclusion of the 20 exercise sessions (post-intervention, week 4), and four weeks after the completion of all exercise sessions (follow-up, week 8). An outcome assessor who was unaware of group allocation performed all outcome measurements.

The participants’ spinal range of motion (ROM) was assessed with a SpinalMouse^®^ device (Idiag AG, Fehraltorf, Sweden). This non-invasive device is used to assess spinal mobility, the reliability of which has been confirmed by other researchers [17,18,19]. Within the scope of this study, the lumbar spine was measured in the sagittal plane. The device measured flexion and extension ROM, comprising the total mobility from maximum flexion to maximum extension. Three measurements were taken, and the mean value was used for statistical analyses. During the test, each subject was asked to stand with their feet placed on marked spots so that the distance between the feet was slightly smaller than the width of the pelvis. Then, the subject made an active movement over a range deemed by them to be maximal while keeping their balance and not using their upper limbs for support.

To quantify disability associated with low back pain, the Oswestry Disability Index (ODI) was used. The ODI is a validated and reliable assessment tool that is suited for use in clinical practice. The results are measured on a 100-point scale (range, 0–100), with higher scores indicating greater dysfunction [20].

The straight leg raise (SLR) test is considered by researchers to be sensitive and specific in diagnosing damage to the peripheral nervous system [21]. The subject’s lower limb was passively raised with the knee extended until potential symptoms were triggered, although not to an angle greater than 60° of flexion in the hip joint, which, according to Kapandji, causes maximum stretching of nerve structures. This test was considered to be positive (1) when it replicated the participant’s symptoms or provoked pain.

The passive lumbar extension (PLE) test was used as a tool for assessing the instability in the lumbar section of the spine [22]. Ferrari et al. suggest that the PLE test is the most suitable test for detecting lumbar instability thanks to its excellent diagnostic accuracy and good reliability [22]. This test was scored as positive (1) when it replicated the symptoms or provoked pain. The procedure was as follows: patient was in the prone position, both legs were then elevated concurrently to a height of about 30 cm from the bed while maintaining the knees extended and pulling the legs [23].

The subjective level of pain associated with the patient’s current condition was assessed based on a numeric rating scale (NRS). The intraclass correlation coefficient of the NRS is 0.95 [24].

### 2.4. Data Analysis

All statistical calculations were performed using the Statistica 12.5 software (StatSoft, Palo Alto, CA, USA). The data were presented as the means and standard deviations (SD) or the medians and interquartile ranges (IQR). The groups were compared at the baseline using a *t*-test for independent samples (unpaired) for continuous variables and the chi-squared test for categorical data. The results of therapy were analyzed using Friedman’s ANOVA with the Wilcoxon post-hoc test. The Friedman’s ANOVA effect size was calculated using Kendall’s W and with interpretation guidelines of 0.1 ≥ x < 0.3 (small effect), 0.3 ≥ x < 0.5 (moderate effect) and x ≥ 0.5 (large effect). For dichotomous values, (SLR/PLE), the Cochran’s Q test was used. Values of *α* < 0.05 were considered to be statistically significant.

## 3. Results

**Figure 1:**
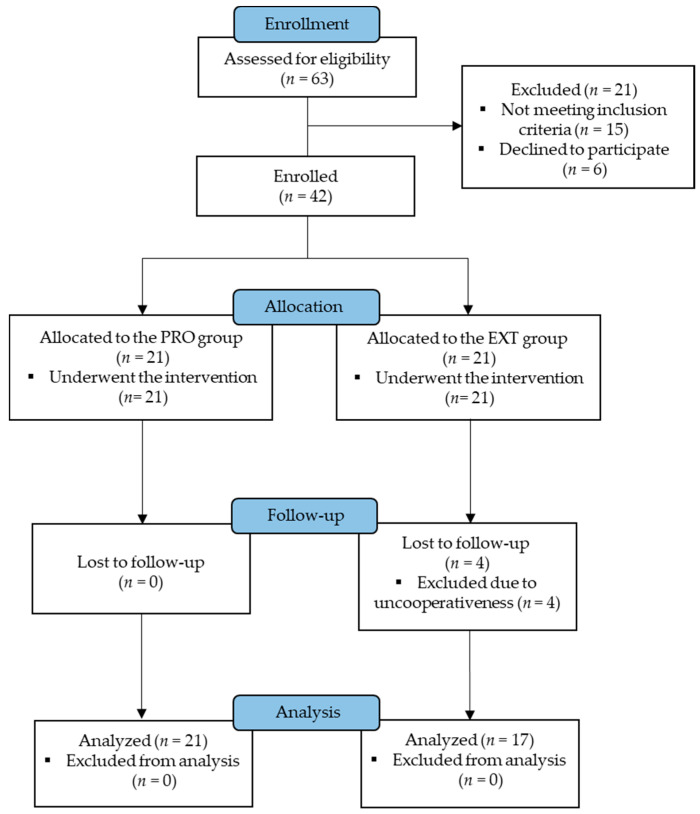
The flowchart of the trial according to the Consolidated Standards of Reporting Trials (CONSORT, non-randomized).

Four participants from the EXT group did not complete the intervention program. The sample size at each assessment timepoint is presented in Figure 1. In total, the study comprised 21 subjects with protrusion (PRO) and 17 subjects with extrusion (EXT) with the average age of 28.24 (SD, 3.88) and 30.17 (SD, 3.30) years, respectively (Table 1). The pre-intervention, post-intervention and follow-up scores for clinical outcomes and tests are presented in Table 2.

In the PRO group, the total range of motion in the sagittal plane from before the intervention to after the intervention and from before the intervention to the follow-up was reduced by 6% in both tests, with values of 88.52° (SD, 6.93), 83.33° (SD, 5.64) and 82.85° (SD, 3.69) measured in the pre-intervention, post-intervention and follow-up assessments, respectively (Kendall’s W = 0.26; *p* = 0.01). The ODI results revealed a reduction post-intervention by 59%, from 16.14 (SD, 6.71) to 6.57 (SD, 0.58) points, and a reduction by 42% (to 9.42 points, SD, 7.15) at the follow-up examination (Kendall’s W = 0.60; *p* < 0.01). The subjective level of pain reported by the participants was reduced by 47% and 65% at the post-intervention and follow-up examinations, respectively (Kendall’s W = 0.86; *p* < 0.01).

In the EXT group, the ROM decreased by 5% and 4% at the post-intervention and follow-up examinations, respectively (pre-intervention, 88.52°; SD, 6.93; post-intervention, 83.33°; SD, 5.64; follow-up, 82.85°; SD, 3.69; Kendall’s W = 0.20; *p* = 0.01). The ODI results were 32% lower after the intervention and 35% lower at the follow-up examination (Kendall’s W = 0.48; *p* < 0.001). The subjective pain level measured using the NRS was decreased by 46% (to 4.85 points, SD, 1.01 vs. to 2.57 points, SD, 1.32) post-intervention and by 55% at the follow-up examination (Kendall’s W = 0.78; *p* < 0.001).

## 4. Discussion

Despite an abundance of previous research, the treatment of DDD still raises considerable uncertainties concerning the course of rehabilitation [25]. The literature comprises numerous publications on the influence and effectiveness of stabilizing exercises for the lumbar multifidus muscle and the transverse abdominal muscle in patients with low back pain [7,26,27,28,29]; however, there is little scientific evidence to support this form of exercise for treating people under 35 years of age. The present study examined another criterion for the division of patients, namely, the type of damage to the intervertebral disc (disc protrusion or extrusion in the subacute stage of the disease according to the ASNR classification). Although Rasmussen–Barr et al. demonstrated the effectiveness of stabilizing exercise in people suffering from subacute DDD, they did not explicitly divide subjects by the type of damage to the intervertebral disc [30]. Preliminary research suggests that the patients’ functional condition varies depending on the progression of the disease. Thus, there are important differences concerning the types of everyday activities and the level of associated pain, where patients with an extruded disc experience greater levels of pain [5].

After four weeks of therapy, the ROM decreased in both groups. During the follow-up period, the tendency for reduced lumbar spine mobility was sustained, although the patients were instructed not to perform any other form of treatment except for the previously administered intervention. This reduced ROM also affected the PLE test results and was probably due to improved neuromuscular control in the area of the muscles stabilizing the lower spine and the general influence of being aware of participating in a research study [31]. When attempting to characterize the biomechanics of the lumbar spine in patients with DDD, Hueng et al. observed a decrease in mobility in the sagittal plane as the pathology progresses [32]. It is also worth bearing in mind that the degeneration of the intervertebral disc could trigger a pathological cycle, the progression of which leads to increasingly limited mobility of vertebral segments [33].

The experience of pain in people suffering from DDD is the main reason for the commencement of treatment, and pain usually accompanies the patient throughout the course of the disease. In the presented research, pain relief was observed in patients both with protruded and extruded discs. Many studies have demonstrated the effectiveness of stabilizing exercise in terms of the level of pain and assessment of the degree of disability [8,34,35], but none have assessed participants based on the progression of disc disease, and the available scientific evidence is often insufficient [25]. The ODI analysis performed in the current study demonstrated the overall effectiveness of stabilizing exercise in both groups. However, because the initial parameters showed statistically significant differences between the groups, interpretation of subsequent stages of the study with the Oswestry questionnaire was not performed due to the non-homogenous nature of the groups. In spite of this, significant differences were observed between the pre-intervention and the post-intervention examinations in both groups. Similar conclusions have been drawn by other researchers, although the clinical condition of subjects was often not determined on the basis of medical imaging [35,36].

When studying subjects with DDD, it is recommended that the irritation of nerve structures be assessed. In this study, we used the SLR. The initial values differed significantly between the two groups. In the PRO group, the results deteriorated; therefore, the therapy did not prove to be successful with respect to the SLR test. However, in the EXT group, a decrease in irritability of nerve structures was observed. In that group, the effect was observed across all stages of the study, although the follow-up results did not differ significantly from the post-intervention examination.

Three factors limit the generalizability of this study. The first is the lack of a control group that received no intervention for disc protrusion and extrusion. For this reason, we could not perform randomization, which severely limits our ability to draw conclusions. Second, as this was a pilot study, the sample size was relatively small. Third, our study did not examine the longitudinal effects of the interventions used; therefore, future research should include follow-up outcome measures. Lastly, in our research, we did not meet the assumptions for parametric tests; therefore, nonparametric tests were used, which are statistically weaker. However, our results may aid in the design of future studies with regard to the study protocol or the generation of effect sizes, which can be used to power a larger randomized clinical trial.

## 5. Conclusions

To date, this study is one of the few to examine the effects of core stability exercise in individuals with DDD, with a distinction between the degree of progression into protrusion and extrusion. Our research showed that stabilization training can reduce pain, improve the clinical condition and daily living activities of people with DDD. However, due to the limitations of our study, the generalizability is limited. Based on the obtained results, we are unable to unambiguously state whether the type of damage to the intervertebral disc significantly affects the results of a stabilizing exercise-based treatment.

## Figures and Tables

**Table 1:** Subjects’ demographic and clinical characteristics.

Variable	PRO (*n* = 21)	EXT (*n* = 17)	*p*
Age (years)	28.24 (3.88)	30.17 (3.30)	0.11
Sex (female/male)	12/9	8/9	0.54
Body mass (kg)	69.85 (15.36)	79.41 (14.62)	0.06
Body height (cm)	172.80 (10.95)	175.05 (12.46)	0.55
BMI (kg/cm^2^)	23.06 (2.02)	25.91 (4.35)	0.01
ROM (^o^)	88.52 (6.93)	81.00 (4.01)	0.00
ODI (0–100)	16.14 (6.7)	22.58 (12.14)	0.04
NRS (0–10)	4.85 (1.01)	5.35 (2.67)	0.43
PLE (%, n)	85.71 (18)	88.23 (15)	0.40 *
SLR (%, n)	0.00 (0)	64.70 (11)	-

BMI—body mass index; ROM—range of motion; ODI—Oswestry Disability Index; NRS—numeric rating scale; PLE—passive lumbar extension test; SLR—straight leg raise test; PRO—protrusion group; EXT—extrusion group; values are expressed as % and *n* or means (SD); * according to the chi-squared test.

**Table 2:** Changes of the clinical outcomes after the intervention.

		PRO (*n* = 21)				EXT (*n* = 17)			
		Pre	Post	Follow-Up	*p*	Pre	Post	Follow-Up	*p*
ROM (^o^)	Mean (SD)	88.52 (6.93)	83.33 (5.64) *	82.85 (3.69) *	0.01	81.00 (4.01)	77.05 (4.74) *	77.94 (6.80) *	0.03
	Median (IQR)	92 (78–94)	86 (80–88)	81 (81–86)		80 (77–84)	77 (73–78)	77 (73–83)	
ODI (0–100)	Mean (SD)	16.14 (6.71)	6.57 (3.58) *	9.42 (7.15) *	<0.01	22.58 (12.14)	15.41 (10.48) *	14.70 (8.18) *	<0.01
	Median (IQR)	14 (10–24)	6 (2–10)	6 (4–14)		18 (12–32)	10 (6–22)	12 (8–20)	
NRS (0–10)	Mean (SD)	4.85 (1.01)	2.57 (1.32) *	1.71 (1.18) *^,^^	<0.01	5.35 (2.66)	2.94 (1.59) *	2.41 (1.69) *^,^^	<0.01
	Median (IQR)	5 (4–6)	3.0 (1–3)	1.0 (1–3)		5 (3–8)	3.00 (2.0–4.0)	2.0 (1.00–4.0)	
PLE	% (n)	85.71 (18)	28.57 (6)	14.29 (3)	<0.01	88.23 (15)	64.70 (11)	17.65 (3)	<0.01
SLR	% (n)	0.00 (0)	14.29 (3)	14.29 (3)	0.04	64.70 (11)	23.53 (4)	11.76 (2)	<0.01

PRO—protrusion group; EXT—extrusion group; ROM—range of motion (total mobility from maximal flexion to maximal extension); ODI—Oswestry Disability Index; NRS—numeric rating scale; PLE—passive lumbar extension test; SLR—straight leg raise test; * statistically significant results in comparison to test 1 according to the Wilcoxson’s post-hoc test; ^ statistically significant results in comparison to test 2 according to the Wilcoxson’s post-hoc test.

## Data Availability

The data presented in this study are available on request from the corresponding author.
